# Genomic Insights into the Giant Shank Phenotype of Dong Tao Chickens Reveal Candidate Loci Involved in Skeletal Growth and Development

**DOI:** 10.3390/genes17080855

**Published:** 2026-07-24

**Authors:** Thuy Thi-Dieu Nguyen, Mauro Penasa, Thuy Thi-Ngoc Dinh, Filippo Cendron

**Affiliations:** 1 Institute of Biology, Vietnam Academy of Science and Technology, 18 Hoang Quoc Viet, Nghia Do, Hanoi 11307, Vietnam; thuy81ibt@gmail.com; 2 Department of Agronomy, Food, Natural Resources, Animals and Environment, University of Padova, 35020 Legnaro, Italy; mauro.penasa@unipd.it

**Keywords:** Dong Tao chicken, genome-wide association study (GWAS), giant shank phenotype, candidate gene, SNPs

## Abstract

Background: The Dong Tao (DT) chicken is a traditional Vietnamese breed characterized by extremely enlarged shanks, representing one of the most distinctive morphological traits observed in domestic chickens. Despite its economic and cultural importance, the genetic basis underlying this phenotype remains poorly understood. Therefore, this study aimed to identify genomic regions and candidate genes associated with the giant shank phenotype in DT chickens. Methods: A total of 87 chickens were analyzed, including 30 DT individuals displaying the giant shank phenotype and 57 control animals from the Ac, Choi, and Ho Vietnamese breeds. Genotype data were obtained from high-density 600K single-nucleotide polymorphism arrays and low-coverage whole-genome sequencing datasets. Genome-wide association study (GWAS) was performed using a linear mixed model, while genetic differentiation between DT and control populations was evaluated through F_ST_ estimates. Candidate regions were further investigated through linkage disequilibrium analyses, gene annotation, and comparison with previously reported chicken quantitative trait loci (QTLs). Results: Four significant genomic regions were identified on chromosomes 2, 4, 5, and 7. These regions harbored several biologically relevant candidate genes, including *IGFBP1*, *IGFBP3*, *MYLK*, *CD44*, and *KALRN*, which are involved in growth regulation, skeletal development, tissue organization, and cellular signaling. The strongest evidence was observed for the chromosome 2 locus containing *IGFBP1* and *IGFBP3*, which exhibited the highest level of genetic differentiation between DT and control populations (maximum FST = 0.47) and overlapped a body weight QTL. Additional overlaps with shank length and feed conversion ratio QTLs were identified in other associated regions. Conclusions: These findings provide novel insights into the genetic architecture underlying the giant shank phenotype in domestic chickens and establish a foundation for future studies aimed at identifying the causal variants and molecular mechanisms involved in giant shank development.

## 1. Introduction

Local chicken breeds represent an important component of global animal genetic resources, preserving unique combinations of adaptive, productive and morphological traits that are often absent or strongly reduced in highly selected commercial lines [1,2,3]. The domestic chicken (*Gallus gallus*) has a complex domestication history rooted in Southeast and South Asia, with genomic studies showing contributions from red jungle-fowl lineages [1,2]. This evolutionary background, together with local breeding traditions and heterogeneous production environments, has generated a wide range of phenotypic diversity among indigenous chicken populations, including variation in body size, growth rate, feathering, behavior, disease resistance, and limb morphology [1,2,3,4]. Characterizing this diversity at the genomic level is essential both for understanding the biological basis of breed-specific traits and for designing conservation strategies that maintain locally adapted genetic resources.

Among Asian indigenous chickens, Vietnamese local breeds are particularly relevant because they combine cultural importance, adaptation to traditional farming systems, and distinctive phenotypic features. The Dong Tao chicken (DT) is one of the most recognizable Vietnamese breeds, mainly because of its extremely enlarged shanks, which represent the hallmark of the breed and contribute strongly to its cultural and economic value [5]. This unusual “giant shank” phenotype makes DT an exceptional biological model for investigating the genomic architecture of limb morphology and tissue overgrowth in chickens [5]. However, despite its distinctive appearance, the genomic basis of the enlarged shank phenotype remains poorly understood. Previous genomic work on Vietnamese chickens has provided valuable information on genetic diversity and structural variation, including a recent genome-wide copy number variation analysis that identified CNV regions containing genes involved in developmental, adaptive, and morpho-functional processes [5,6].

Morphological and growth-related traits in chickens are typically polygenic and influenced by several biological processes, including skeletal development, muscle growth, endocrine regulation, extracellular matrix organization, and cellular signaling [6,7,8,9,10]. Quantitative trait locus (QTL) mapping and a genome-wide association study (GWAS) have shown that body weight, body size, shank length, and shank width are controlled by multiple genomic regions distributed across the genome [6,7,8,9]. For example, QTL mapping in chicken lines divergently selected for body weight identified loci affecting both growth and shank traits [7], while recent GWAS in local and commercial chicken populations have identified genomic regions associated with body weight, shank length, shank width, and other conformation traits [7,8,9]. These findings support the idea that extreme limb-related phenotypes, such as the DT giant shank, are unlikely to be explained by a single locus and are more plausibly influenced by a set of genomic regions affecting growth regulation and tissue development [5].

The insulin-like growth factor (IGF) system is one of the most relevant biological pathways for growth and body-size variation in chickens [10]. IGF signaling contributes to muscle development, post-hatch growth, nutrient partitioning, and systemic growth regulation [10,11]. In birds, IGF-related genes and IGF-binding proteins are involved in the modulation of growth rate and muscle accretion, and variation in the somatotropic axis has been linked to differences in body size and performance [10,11,12]. In this context, genes such as *IGFBP1* and *IGFBP3* are particularly interesting candidates because they regulate IGF bioavailability and may influence tissue growth and skeletal development. In addition to endocrine regulation, genes involved in cytoskeletal organization, muscle contraction, and cell adhesion may contribute to limb morphology by modulating tissue architecture, cellular remodeling, and local growth dynamics. For instance, *MYLK* is involved in myosin light-chain phosphorylation and muscle-related contractile processes, while *TNS3* encodes a focal adhesion-associated protein involved in cytoskeletal organization and cell–matrix interactions [13,14]. These biological functions are highly relevant when considering a phenotype characterized by extreme enlargement of the lower limb region.

To facilitate the biological interpretation of GWAS signals associated with the giant shank phenotype, candidate genomic regions can be integrated with functional gene annotation and previously reported chicken QTLs related to skeletal growth and body development. These complementary analyses help prioritize biologically plausible candidate genes and genomic regions for further investigation [15,16,17,18].

In the present study, we performed a GWAS to identify genomic regions associated with the giant shank phenotype in DT chickens compared with Vietnamese control breeds. Candidate regions were annotated to identify protein-coding genes located within the genomic windows surrounding the lead single nucleotide polymorphisms (SNPs). To strengthen the biological interpretation of the results, GWAS candidate regions were further integrated with QTL overlap analysis, population differentiation estimates, and functional prioritization of candidate genes. The aim of this work was to provide the first genomic focus of the giant shank phenotype in DT chickens and to identify candidate loci and genes potentially involved in growth regulation, limb morphology, and tissue development.

## 2. Materials and Methods

### 2.1. Animals, Phenotypic Classification, and Genomic Data Sources

**Figure 1:**
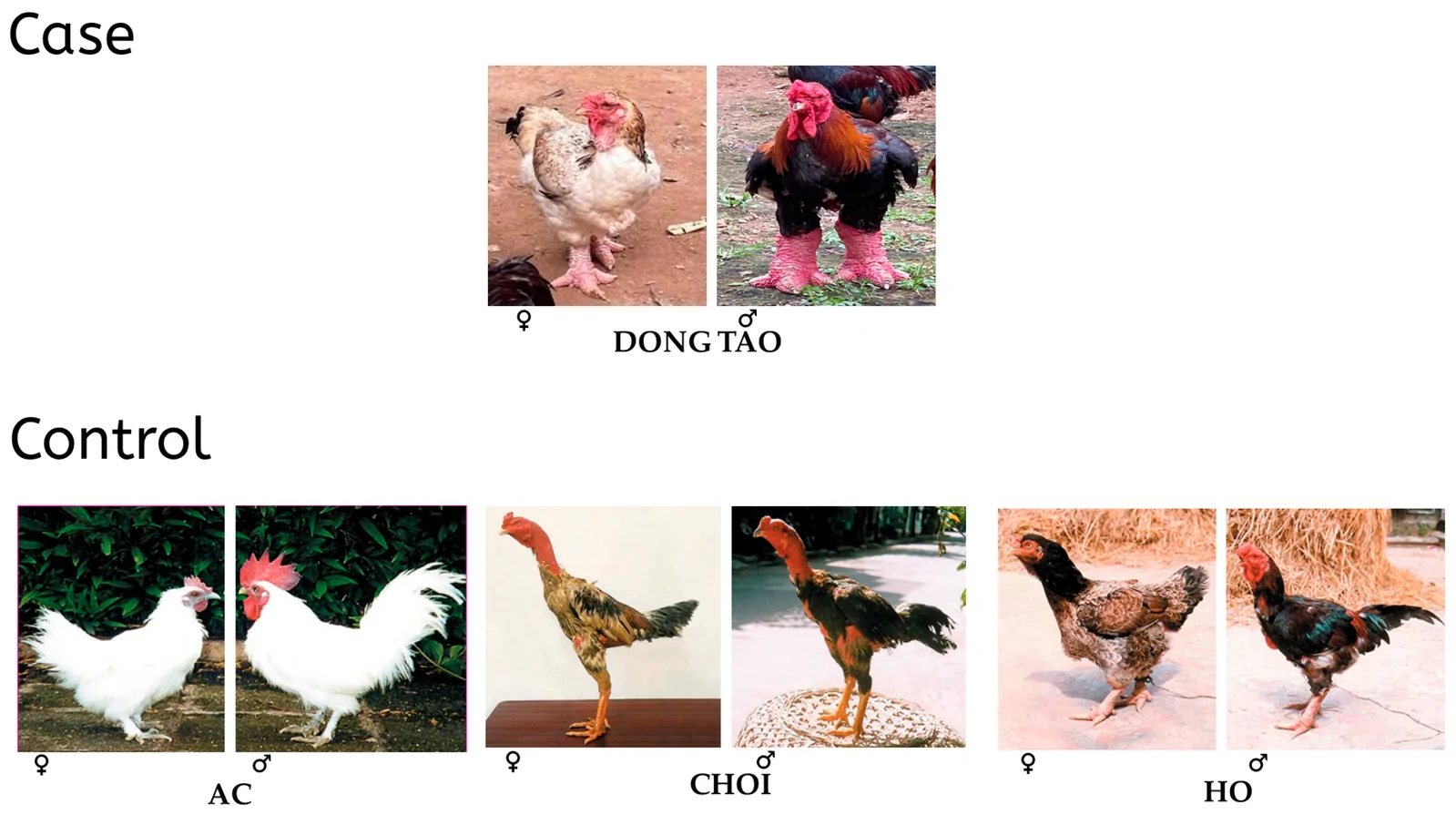
Representative shank phenotypes of the case and control populations included in the study.

A total of 87 chickens belonging to four Vietnamese local breeds were included in this study. The case group consisted of 30 animals belonging to DT breed, which is characterized by a markedly enlarged shank phenotype. The control group comprised 57 individuals from Ac (AC), Choi (CI), and Ho (HO) breeds, all displaying a different shank morphology. Phenotypes were coded as a binary trait, with DT chickens classified as cases and AC, CI, and HO classified as controls (Figure 1). Genomic data were obtained from two complementary sources. A total of 18 DT chickens and all 57 control individuals were retrieved from the SYNBREED chicken diversity panel and had been previously genotyped using the Affymetrix Axiom^®^ Genome-Wide Chicken Genotyping Array (Thermo Fisher Scientific, Waltham, MA, USA), which includes approximately 580,000 SNPs distributed across the chicken genome [19].

To increase the representation of the DT population, an additional 12 DT chickens were included using low-coverage whole-genome sequencing data which originated from a previous genomic investigation of Vietnamese local chickens [5]. Raw sequencing reads were aligned against the *G. gallus* reference genome assembly GRCg6a (Ensembl ID: GCA_000002315.5) using Bowtie 2 v2.5.5 [20]. Although DT chickens represent a highly specialized domestic breed, candidate gene annotation relied on the Red Junglefowl-derived reference genome, ensuring high-quality gene models and evolutionary consistency.

Alignment files were generated in Sequence Alignment/Map format and subsequently processed to extract genomic coordinates corresponding to aligned reads. Because sequencing coverage was limited, *de novo* variant discovery was not performed. Instead, sequencing information was used to recover genotypes at predefined SNP positions corresponding to markers present on the high-density SNP array.

### 2.2. SNP Identification, Dataset Integration, and Quality Control

To construct a common genotype dataset across the two data sources, the SNP-array marker positions were used as the reference coordinate set. Marker coordinates were extracted from the PLINK BIM file and converted into BED format by retaining chromosome, start, and end positions for each SNP [21]. Sequencing-derived genomic positions were processed in the same coordinate format, and BEDTools v2.31.1 intersect was used to identify loci shared between the sequencing-derived dataset and the SNP-array marker set [22]. Only loci overlapping the SNP-array marker coordinates were retained from the sequencing-derived data. This step was applied to avoid independent variant discovery from low-coverage sequencing data and to restrict the final dataset to a predefined set of array-based SNP markers. The resulting sequencing-derived genotypes were therefore represented only at positions directly comparable with the SNP-array dataset. Variant calling from the low-coverage whole-genome sequencing data was performed using BCFtools v1.9 [23]. Genotype likelihoods were generated using bcftools mpileup against the *G. gallus* GRCg6a reference genome, followed by variant calling with the multiallelic model implemented in bcftools call -m. Default mpileup settings were used, including a minimum base quality of Phred 13. Only variant sites were emitted by the caller. The resulting VCF was subsequently restricted to genomic positions corresponding to markers included in the high-density SNP array using bcftools view -R. No genotype imputation was performed, and genotypes that could not be assigned by the caller were retained as missing values. Following allele harmonization and dataset merging, variants with unresolved allele or strand inconsistencies were excluded.

The SNP-array and sequencing-derived genotype datasets were then merged using PLINK v1.9 [21]. During merging, variants with strand inconsistencies were identified from PLINK merge reports. Allele flipping was performed when inconsistencies were attributable to strand orientation, whereas variants showing unresolved allele mismatches or ambiguous allele coding were removed.

Quality control was performed on the merged dataset using PLINK v1.9 [21]. The SNPs with call rate <95% and minor allele frequencies <5%, and animals with more than 10% of missing genotypes were discarded from the dataset [16]. Finally, markers located on unplaced scaffolds, random contigs, or poorly characterized genomic regions were excluded, retaining only SNPs assigned to assembled chicken chromosomes. After quality control, a total of 166,152 SNPs remained for subsequent analyses. The final quality-controlled dataset was used for population structure assessment, GWAS, and downstream candidate-region annotation.

### 2.3. Population Structure Analysis

To investigate the genetic relationships among individuals and assess potential population stratification, linkage disequilibrium (LD) pruning was performed using PLINK v1.9 [21] with the command --indep-pairwise 50 5 0.2. This procedure removed highly correlated markers using a sliding window of 50 SNPs, advanced by 5 SNPs at each step, and retained variants with pairwise LD values below r^2^ = 0.20. The pruned marker set was subsequently used to perform multidimensional scaling (MDS) analysis in PLINK v1.9 [21]. The first two MDS dimensions were visualized to evaluate genetic clustering patterns among DT chickens and the three Vietnamese control populations (AC, CI and HO), as well as to identify potential outliers and population substructure that could influence downstream association analyses.

### 2.4. Genome-Wide Association Analysis

Genome-wide association analyses were performed using GEMMA v0.98 [24]. GEMMA explicitly models the genomic relationship matrix, thereby reducing spurious associations generated by population structure and cryptic relatedness. To account for relatedness among individuals and residual population structure, a centered genomic relationship matrix was first estimated from genome-wide SNP data using the -gk 1 option implemented in GEMMA. Association testing was subsequently conducted using a linear mixed model in which the genomic relationship matrix was incorporated as a random effect. The phenotype was analyzed as a binary trait, with DT chickens classified as cases and the remaining Vietnamese populations as controls. Statistical significance was assessed using likelihood ratio test [LRT, −log_10_(P)] statistics generated by GEMMA. To account for multiple testing, a genome-wide significance threshold was calculated using Bonferroni correction based on the number of SNPs included in the final quality-controlled dataset. Manhattan and quantile–quantile plots were generated in R v4.4.0 [25] to visualize association signals and evaluate the distribution of test statistics. All graphical representations were performed in R.

### 2.5. Pairwise Association Analyses and Genetic Differentiation

To further investigate population-specific association signals, three additional GWAS analyses were conducted by comparing DT chickens separately against each control population. The same linear mixed model framework implemented in GEMMA v0.98 [24] was applied in all pairwise comparisons. Genetic differentiation between DT chickens and the pooled control populations was assessed through SNP-wise estimation of Weir and Cockerham’s F_ST_ [26] using PLINK v1.9 [21]. Individuals were assigned to two groups (DT and controls), and F_ST_ values were calculated for all SNPs passing quality control filters. For each candidate GWAS region, summary statistics including mean F_ST_, median F_ST_, maximum F_ST_, and the highest-ranked differentiated SNP were subsequently calculated. This analysis was used as complementary evidence to evaluate whether genomic regions associated with the enlarged shank phenotype displayed elevated allele-frequency divergence between DT chickens and the control populations.

### 2.6. Annotation of Candidate Regions and QTL Co-Localization

Candidate genomic regions were defined around the most significant GWAS signals. For each lead SNP, a genomic interval extending 500 kb upstream and 500 kb downstream was considered, generating a 1-Mb candidate region centered on the associated marker. Gene annotation was performed using the *G. gallus* reference genome assembly GRCg6a and the corresponding Ensembl gene annotation release. Genomic coordinates of candidate regions were intersected with annotated gene positions using BEDTools v2.31.1 [22]. Both genes directly overlapping the associated intervals and genes located in close proximity to the lead SNP were retained for further investigation.

Positional candidate genes identified within associated regions were ranked according to their physical proximity to the lead SNP and their known biological functions. Particular attention was given to genes previously implicated in skeletal development, limb morphogenesis, growth regulation, cartilage formation, bone metabolism, and developmental signalling pathways. To provide additional biological support for candidate regions, associated intervals were compared with QTLs reported in the Animal QTL Database (Chicken QTLdb: https://www.animalgenome.org/cgi-bin/QTLdb/GG/index, accessed on 1 May 2026) [27]. Genomic coordinates of candidate regions were intersected with published QTL intervals using BEDTools v2.31.1 [22]. Emphasis was placed on overlaps involving growth-related traits, including body weight, shank length, skeletal development, and feed efficiency.

## 3. Results

### 3.1. Population Structure and Genome-Wide Association Analysis

**Figure 2:**
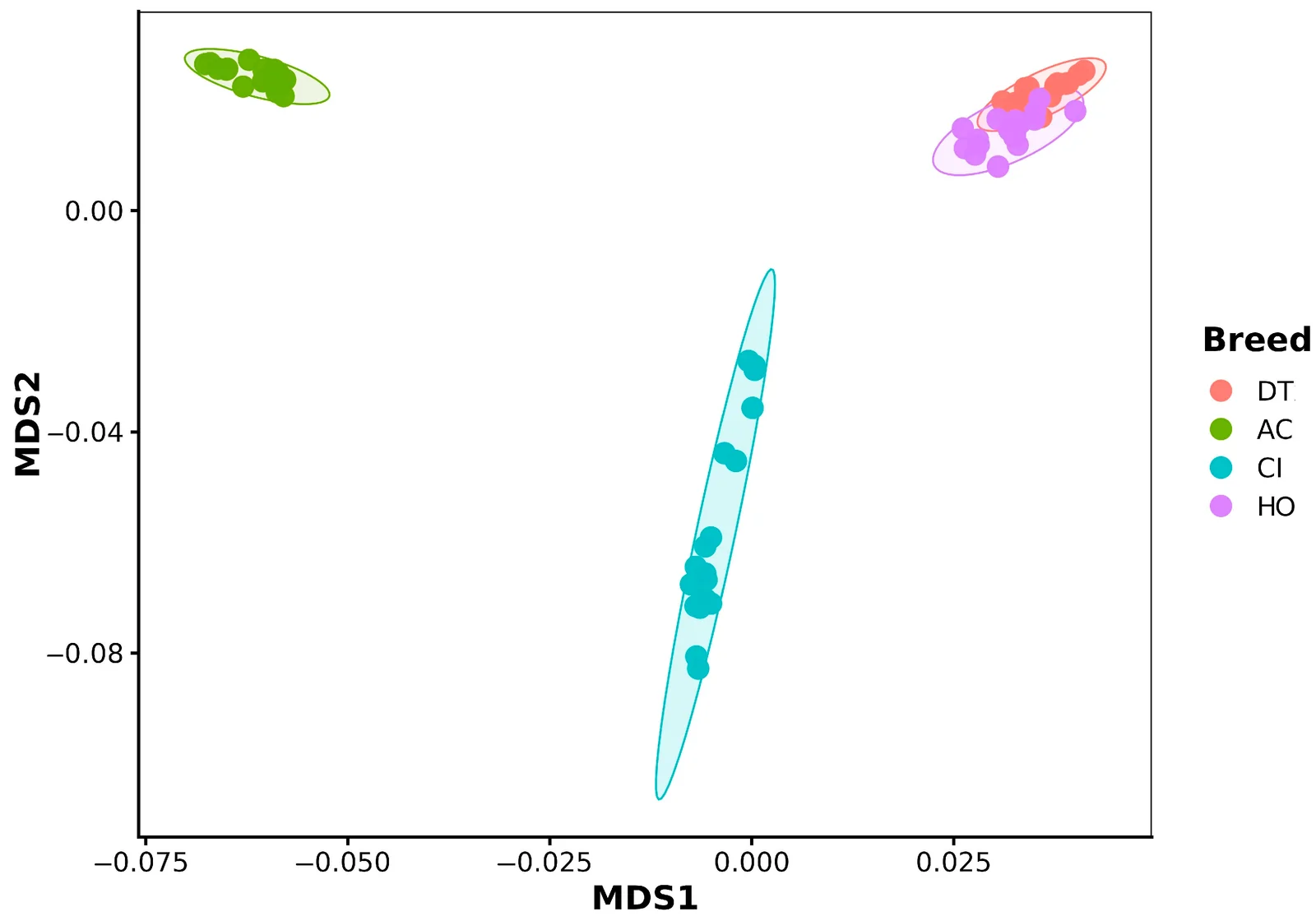
Multidimensional scaling plot based on genome-wide SNP data showing the genetic relationships among the four Vietnamese chicken populations. Each point represents an animal.

Multidimensional scaling analysis performed on the LD-pruned dataset revealed a clear genetic separation among the four Vietnamese chicken populations included in this study (Figure 2). Individuals clustered according to breed assignment, with DT chickens forming a distinct group separated from the AC, CI, and HO populations. AC and H individuals occupied neighboring positions along the first MDS axis, whereas CI chickens formed an independent cluster primarily separated along the second dimension. No overlap between DT and control populations was observed.

The distribution of GWAS test statistics was evaluated using a quantile–quantile plot (Figure S1). Observed and expected distributions were largely concordant across most markers, while deviations from the null expectation were restricted to the upper tail of the distribution. The genomic inflation factor was 0.77, indicating a conservative distribution of the test statistics.

**Figure 3:**
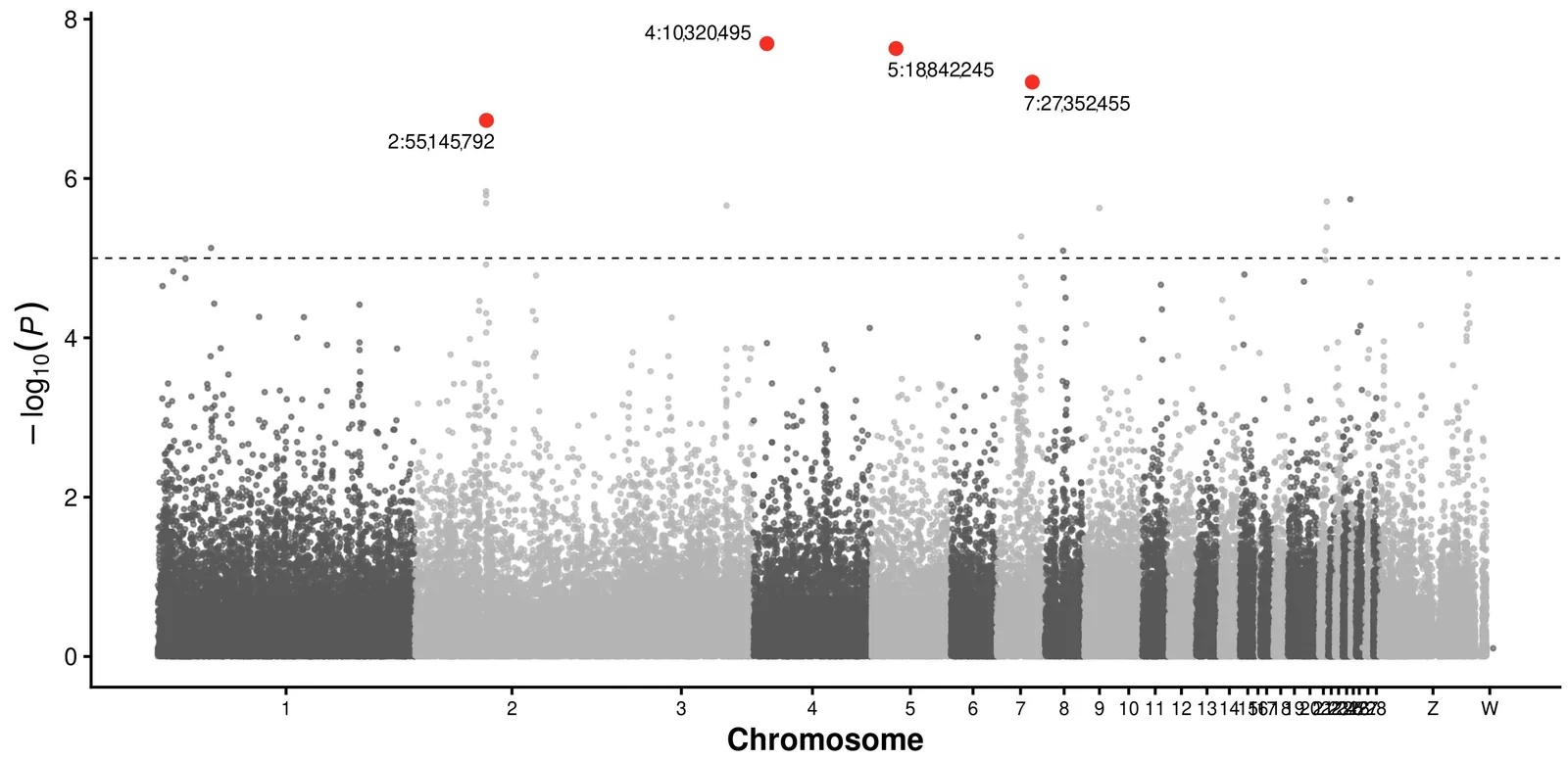
Genome-wide association analysis of the giant shank phenotype in Dong Tao chickens. Four significant association signals were detected on chromosomes 2, 4, 5, and 7, highlighting genomic regions potentially involved in the genetic control of limb growth and shank development. The dashed line indicates the genome-wide significance threshold.

Genome-wide association analysis identified four major genomic regions exceeding the genome-wide significance threshold (Figure 3). The most significant association signal was detected on chromosome 4 at position 10,320,495 bp (*p* = 2.03 × 10^−8^), corresponding to an LRT of 7.69. Additional significant loci were identified on chromosome 5 at 18,842,245 bp [*p* = 2.34 × 10^−8^; LRT = 7.63], chromosome 7 at 27,352,455 bp [*p* = 6.17 × 10^−8^; LRT = 7.21], and chromosome 2 at 55,145,792 bp [*p* = 1.87 × 10^−7^; LRT = 6.73]. The four associated loci were subsequently defined as candidate genomic regions by considering a 1-Mb interval centered on each lead SNP (±500 kb). These regions contained between 79 and 125 SNPs and were selected for downstream annotation, genetic differentiation analyses, and candidate gene prioritization.

### 3.2. Characterization of Candidate Regions

Four genomic regions were identified as significantly associated with the giant shank phenotype in DT chickens (Table 1). The lead SNPs were located on chromosomes 2, 4, 5, and 7, and were subsequently used to define candidate intervals extending ±500 kb around each association peak.

The candidate regions varied in size and gene content. Region 1 on chromosome 4 extended from 9.82 to 10.82 Mb and contained three annotated genes (*SLITRK4*, *GABRQ*, and *GABRA3*). Region 2 on chromosome 5 covered the interval between 18.34 and 19.34 Mb and contained twenty annotated genes, including *SHANK2*, *FBXO3*, *NAT10*, *CAT*, *ELF5*, *EHF*, *APIP*, *CD44*, and *SLC1A2*. Region 3 on chromosome 7 encompassed eight annotated genes, including *MYLK*, *KALRN*, *ADCY6*, *HACD2*, *CCDC14*, *SEC22A*, *PDIA5* and ENSGALG00000050781. Region 4 on chromosome 2 contained fifteen annotated genes, including *IGFBP1*, *IGFBP3*, *ADCY1*, *TNS3*, *SLC12A7*, *TRIP13*, *NFX1*, and *TPPP*.

**Figure 4:**
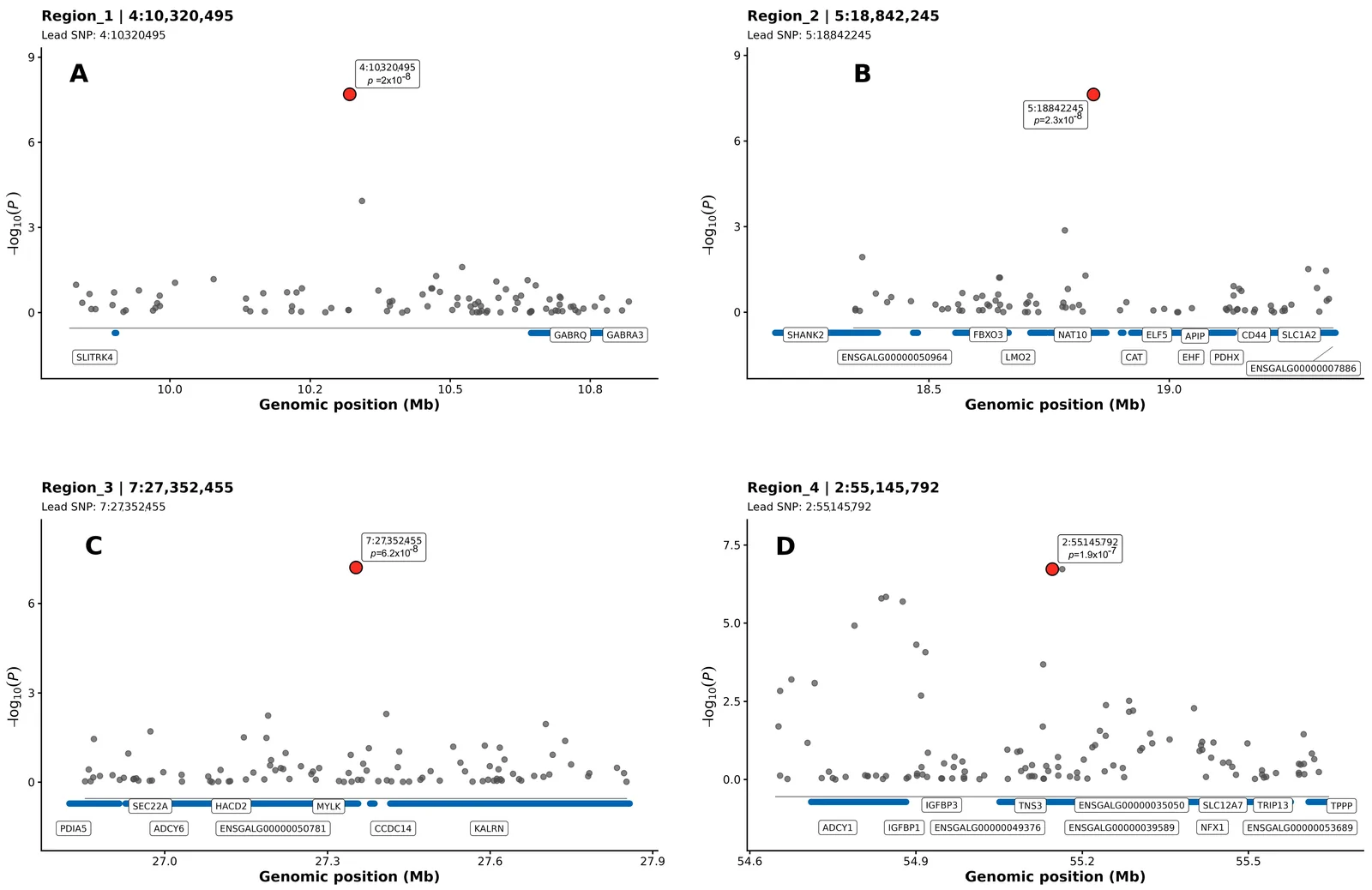
Detailed view of the four candidate loci associated with the giant shank phenotype in Dong Tao chickens. Regional association plots illustrate the distribution of association signals surrounding the most significant SNPs on chromosomes 4 (**A**), 5 (**B**), 7 (**C**), and 2 (**D**).

Regional association plots provided a detailed representation of the four significant loci and their local genomic context (Figure 4). In each region, the lead SNP corresponded to the strongest association signal detected within the interval. The chromosome 4 locus was centered on SNP 4:10,320,495 and mapped within the interval containing *SLITRK4*, *GABRQ*, and *GABRA3* (Figure 4A). The chromosome 5 signal was located near a cluster of genes including *SHANK2*, *CAT*, *ELF5*, *CD44*, and *SLC1A2* (Figure 4B). The chromosome 7 association peak mapped within a region containing *MYLK*, *KALRN*, *ADCY6*, and *HACD2* (Figure 4C), whereas the chromosome 2 locus was located close to *IGFBP1*, *IGFBP3*, *TNS3*, and *ADCY1* (Figure 4D).

The complete list of the top 200 associated SNPs identified in the GWAS analysis is reported in Table S1. Annotation of the four candidate regions identified a total of 46 genes located within the associated intervals (Table S2). Candidate gene prioritization highlighted *IGFBP1* and *IGFBP3* as high-priority positional candidates, whereas the remaining genes were retained as positional candidates based on their genomic proximity to the lead association signals (Table 2).

Functional categorization of all genes identified within the candidate regions is reported in Table S3. The annotated genes were assigned to biological categories including growth regulation, developmental processes, signal transduction, cytoskeletal organization, cell adhesion, and oxidative stress response.

### 3.3. Functional Characterization of Candidate Regions and Supporting Genomic Evidence

To further investigate the biological relevance of the genomic regions identified by the GWAS, LD patterns, population differentiation statistics, previously reported QTLs, and the functional annotation of positional candidate genes were examined.

**Figure 5:**
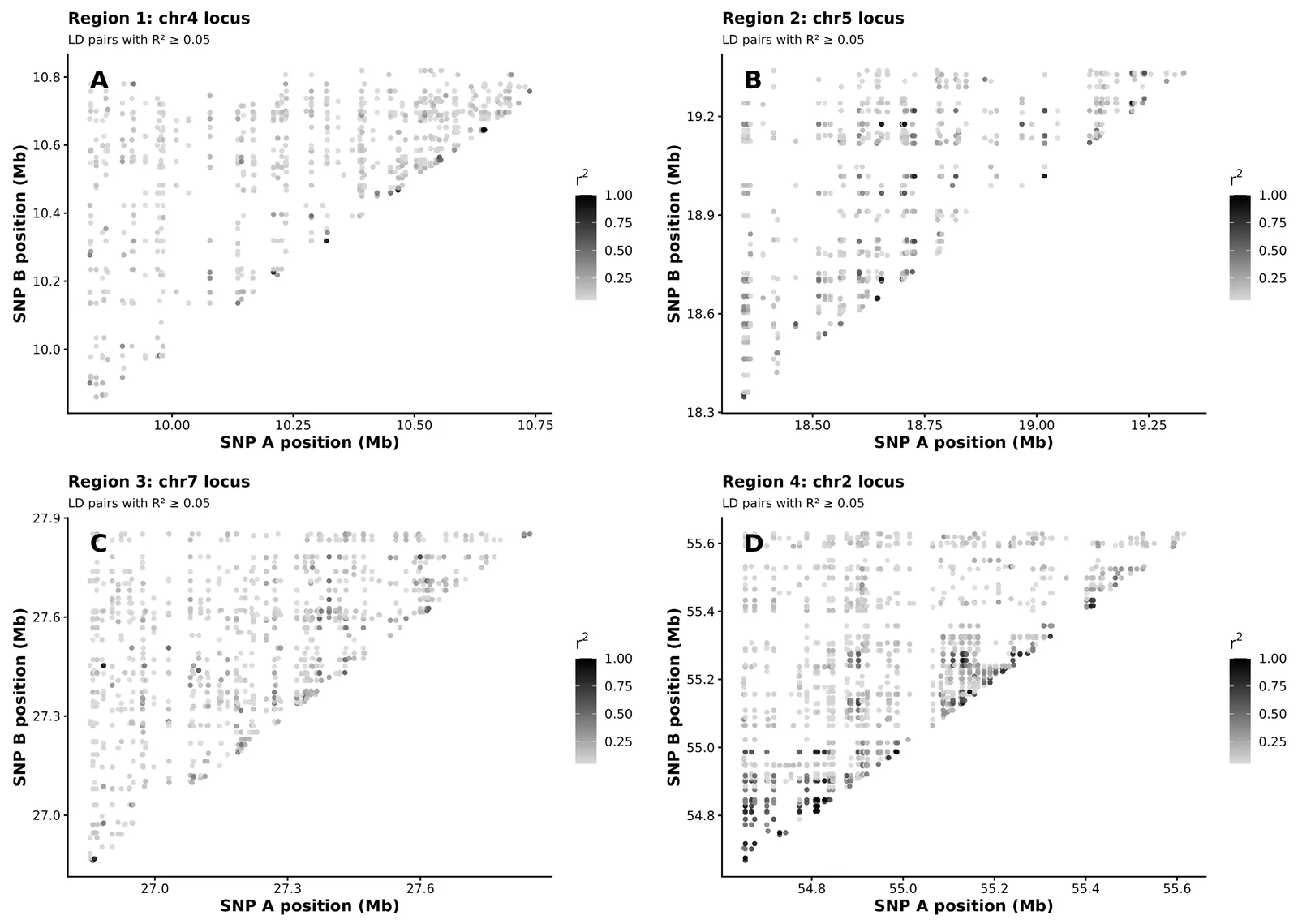
Pairwise linkage disequilibrium (LD) patterns within the four candidate genomic regions. Panels (**A**–**D**) correspond to the loci on chromosomes 4, 5, 7, and 2, respectively. Each point represents a pairwise SNP comparison, with shading indicating the magnitude of linkage disequilibrium (r^2^).

The four candidate regions identified by the GWAS analysis displayed distinct LD architectures (Figure 5). The strongest and most extended LD blocks were observed within the chromosome 2 locus harboring the *IGFBP1–IGFBP3* region and within the chromosome 4 locus, indicating the presence of relatively conserved haplotypes surrounding the associated variants. In contrast, the chromosome 5 and chromosome 7 regions showed a more fragmented LD structure characterized by multiple smaller LD clusters. Analysis of genetic differentiation between DT and control populations revealed elevated F_ST_ values within all four GWAS candidate regions (Table S4; Figure S2). The strongest signal was detected in the chromosome 2 region, which exhibited a maximum F_ST_ value of 0.474 and contained the 34th most differentiated SNP across the entire genome. Additional differentiation peaks were identified on chromosome 4 (maximum F_ST_ = 0.362; genome-wide rank = 262) and chromosome 7 (maximum F_ST_ = 0.319; genome-wide rank = 507), whereas the chromosome 5 region showed a weaker but still detectable signal (maximum F_ST_ = 0.145). These results indicate that several GWAS loci are also characterized by substantial allele frequency divergence between DT chickens and control breeds.

**Figure 6:**
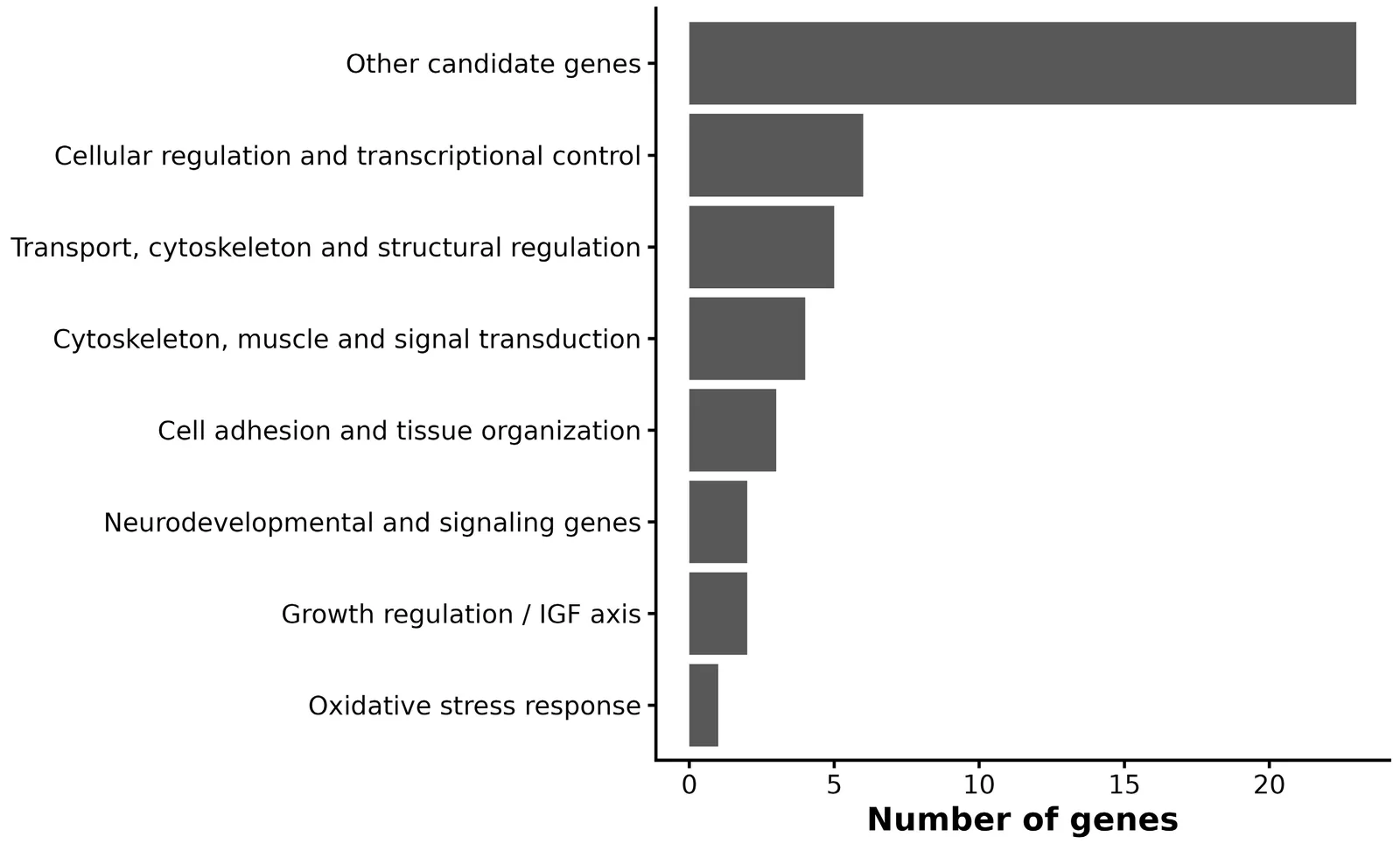
Distribution of candidate genes across major functional categories.

Comparison with the Chicken QTL Database identified multiple previously reported QTLs overlapping the associated regions (Table 3). Notably, the chromosome 4 region overlapped a previously reported shank length QTL, providing additional biological support for the relevance of this candidate region in skeletal morphology. Additional overlaps were detected with QTLs affecting body weight, feed conversion ratio, and egg production traits. In particular, the chromosome 2 region containing *IGFBP1* and *IGFBP3* co-localized with a body weight QTL, whereas the chromosome 5 region overlapped feed conversion ratio QTLs. Overall, five growth-related QTL associations were detected across the candidate regions, supporting the relevance of these loci for growth and morphological development. A total of 46 positional candidate genes were identified within the four associated regions (Table S2). Functional classification of these genes highlighted several biological categories potentially involved in the development of the giant shank phenotype (Figure 6). The functional classification revealed that most positional candidate genes were associated with cellular regulation and structural processes, including cytoskeletal organization, muscle development, cell adhesion, and tissue organization. Smaller functional categories included genes involved in growth regulation through the IGF axis, oxidative stress response, and neurodevelopmental signaling, highlighting the diverse biological pathways potentially contributing to the giant shank phenotype.

Genes related to growth regulation and the IGF axis included *IGFBP1* and *IGFBP3*. Cytoskeletal organization, muscle development, and signal transduction were represented by genes such as *MYLK* and *KALRN*, whereas cell adhesion and tissue organization functions included *TNS3* and *CD44*. Additional candidates were associated with transcriptional regulation, intracellular transport, oxidative stress response, and neurodevelopmental signaling pathways. Collectively, the convergence of GWAS signals, local LD structure, elevated population differentiation, overlap with previously reported QTLs, and the biological functions of positional candidate genes provides multiple complementary lines of evidence supporting the biological plausibility of these genomic regions as candidate loci associated with the giant shank phenotype in DT chickens. However, neither elevated F_ST_ values nor QTL co-localization constitute independent validation of the observed GWAS associations.

## 4. Discussion

### 4.1. Interpretation of GWAS Findings

The DT represents one of the most distinctive indigenous chicken breeds worldwide, primarily due to its characteristic giant shank phenotype. Despite its cultural and economic importance in Vietnam, the genetic mechanisms underlying extreme limb morphology have remained largely unexplored. In the present study, we combined GWAS analysis, population differentiation statistics, LD patterns, QTL information, and candidate gene annotation to investigate the genomic architecture associated with this unique phenotype. Although the present study employed a breed-based case–control design, the control group was not exclusively composed of genetically distant breeds with typical shank morphology. In particular, Ho chickens showed a close genetic relationship with DT in the MDS analysis and are characterized by relatively enlarged shanks. Therefore, the control group included a genetically related population displaying an intermediate shank phenotype rather than exclusively genetically distant breeds with conventional shank morphology.

The GWAS identified four genomic regions located on chromosomes 2, 4, 5, and 7 that were significantly associated with the giant shank phenotype. Importantly, these signals were obtained after accounting for population structure using a mixed-model framework, and the absence of substantial inflation in the association statistics indicated that the identified loci were unlikely to represent spurious associations caused by cryptic relatedness or breed stratification. The identification of a limited number of significant regions is consistent with the possibility that loci with relatively large effects contribute to the giant shank phenotype, although additional loci of smaller effect may have remained undetected because of the relatively low statistical power of the study [2,28,29]. The genomic inflation factor (λ = 0.77) indicated a slight deflation of the test statistics rather than inflation. Such conservative behavior is commonly observed in GWASs performed on relatively small datasets using linear mixed models, where correction for relatedness and population structure can reduce the overall distribution of association statistics [24]. Consequently, while this conservative approach decreases the likelihood of false-positive associations, it may also reduce statistical power and increase the probability of false negatives. Therefore, additional loci contributing to the giant shank phenotype may have remained undetected.

Among the identified loci, the chromosome 2 region appears particularly compelling. This interval contains both *IGFBP1* and *IGFBP3*, members of the IGF binding protein family. The IGF signaling pathway is one of the principal regulators of skeletal growth and developmental processes in vertebrates. Previous studies have demonstrated that *IGF-I* directly regulates longitudinal bone growth, bone mineralization, and overall skeletal development [30,31,32]. Furthermore, IGFBP proteins modulate the bioavailability of IGFs within tissues and therefore play a critical role in regulating growth responses [31,33,34]. Experimental studies have shown that alterations in *IGFBP3* activity influence osteoblast proliferation and bone formation [34], while *IGF-I/IGFBP-3* complexes have been associated with increased cortical bone growth and lean tissue development [35,36]. The localization of both *IGFBP1* and *IGFBP3* within the strongest differentiated candidate region identified in the present study suggests that modulation of the IGF signaling pathway may contribute to the remarkable enlargement of DT shanks. Additional biological evidence comes from the F_ST_ analysis, which showed that this genomic region is highly differentiated between DT and control populations. The chromosome 2 region exhibited the highest degree of differentiation between DT and control populations, with the top SNP ranking among the most differentiated loci genome-wide. Elevated F_ST_ values are commonly interpreted as signatures of population-specific selection [26,37]. Therefore, the observed differentiation is consistent with historical selection acting on this genomic region [5].

The chromosome 7 locus identified in the present study contains the *MYLK* gene, which emerged as one of the most interesting positional candidate genes. *MYLK* encodes myosin light-chain kinase, a central regulator of cytoskeletal organization and cellular contractility. Although *MYLK* has not been previously implicated directly in giant shank development, its involvement in tissue remodeling, cellular migration, and structural organization suggests a plausible role in determining the architecture of skeletal and connective tissues. Interestingly, this region also contains *KALRN* and *ADCY6*, genes involved in intracellular signaling and cytoskeletal regulation. Together, these genes may contribute to the development and maintenance of the enlarged limb structures characteristic of DT chickens.

The chromosome 5 candidate region harbors several positional candidate genes potentially involved in tissue growth and organization, including *CD44*, *SHANK2*, *CAPRIN1*, *CAT*, and *NAT10*. Among these, *CD44* is particularly noteworthy because it mediates interactions between cells and the extracellular matrix, and plays important roles in tissue remodeling and developmental processes [38]. Altered regulation of extracellular matrix organization has been repeatedly associated with changes in skeletal morphology across vertebrate species [39]. Therefore, *CD44* and other genes located within this candidate region represent plausible biological candidates that may contribute to the structural development of DT limbs, although functional studies will be required to clarify their role.

The chromosome 4 region provides additional evidence supporting the validity of the GWAS findings. Most notably, this region overlapped a previously reported shank length QTL [27]. The co-localization of the identified regions with previously reported QTLs provides additional biological support for their potential involvement in the giant shank phenotype, indicating that independent studies have repeatedly highlighted these genomic intervals in relation to growth- and skeletal-related traits. Nevertheless, QTL overlap represents complementary evidence rather than independent replication of the associations identified in the present study. Previous studies have identified multiple genomic regions affecting shank length and skeletal integrity in chickens [40,41,42]. The concordance between our GWAS signal and these previously reported loci substantially strengthens the hypothesis that this region contributes to limb morphology in DT chickens.

The integration of GWAS and QTL information revealed that several candidate regions overlap genomic intervals associated with body weight, feed conversion ratio, and skeletal traits. Such overlaps are biologically meaningful because growth performance and limb development are tightly interconnected processes in poultry. Selection for increased body size frequently influences skeletal characteristics through pleiotropic effects or shared developmental pathways [43,44,45,46]. Indeed, intensive selection for rapid growth in commercial chicken breeds has been associated with accelerated metabolism and an increased incidence of leg and shank disorders. Such conditions often compromise normal skeletal development, leading to weaker limb structures, reduced mechanical integrity, and greater susceptibility to skeletal fragility [47]. Therefore, the giant shank phenotype may represent the outcome of selection acting simultaneously on growth-related and structural developmental mechanisms. The LD analyses further support the robustness of the identified signals. In all four candidate regions, the lead SNPs were embedded within broader LD blocks rather than occurring as isolated markers. This pattern is consistent with the expectation that causative variants are inherited together with neighboring polymorphisms and suggests that the detected associations reflect genuine genomic signals rather than random statistical artifacts. The extensive LD observed in some regions may also reflect the demographic history of DT chickens, including potential founder effects and directional selection [48,49].

The functional classification of candidate genes highlighted several biological processes potentially contributing to the giant shank phenotype, including growth regulation, developmental signaling, cytoskeletal organization, cell adhesion, oxidative stress response, and tissue remodeling. Notably, the coexistence of genes involved in growth factor signaling (*IGFBP1* and *IGFBP3*), cytoskeletal regulation (*MYLK* and *KALRN*), and extracellular matrix interactions (*CD44* and *TNS3*) supports the biological plausibility of these genomic regions as candidates associated with the giant shank phenotype. Rather than representing a simple increase in skeletal size, the DT phenotype likely reflects complex interactions among pathways controlling bone growth, connective tissue expansion, and structural organization [5,7,50,51]. Overall, the functional classification suggests that the phenotype is unlikely to be explained by variation in a single biological pathway. Instead, the positional candidate genes are distributed across functional categories involved in skeletal growth, cytoskeletal organization, tissue architecture, and growth factor signaling, indicating that multiple biological processes may contribute to the development of this distinctive morphological trait. However, these functional interpretations are based on the known biological roles of positional candidate genes, and further functional studies will be required to establish their direct involvement in the giant shank phenotype.

The results of the present study are consistent with previous genome-wide CNV analyses performed in Vietnamese local chickens, which showed that DT harbors a broad structural genomic variability involving genes associated with developmental regulation, skeletal and mesenchymal development, extracellular matrix organization, collagen modification, and tissue remodeling [5]. In that study, CNVRs identified in DT overlapped genes such as *LRP4, ZIC1*, *ZIC4*, *JARID2*, *KMT2C*, *PLOD2*, *OGN*, *OMD*, *ACTA1*, *BGN*, and *CREB3L2*, suggesting that developmental and structural pathways may contribute to the phenotypic differentiation of this breed [5]. Although the candidate genes identified here by a GWAS do not completely overlap with the CNVR-embedded genes reported previously, the findings of the two studies are highly consistent at the functional level. In particular, the present GWAS identified loci harboring *IGFBP1*, *IGFBP3*, *MYLK*, *CD44*, *KALRN*, and *TNS3*, genes involved in growth regulation, cytoskeletal organization, cell adhesion, and tissue remodeling. Similarly, Bai et al. (2026) [6] profiled genome-wide CNVs in DT chickens and nine other local breeds and performed a breed-based CNV-GWAS. They identified a deletion on chromosome 3 (36,529,501–36,539,000 bp) that was specific to DT and may represent a potential marker for breed discrimination. Functional enrichment analysis further identified *TPK1, SOCS2*, and *FHIT* as candidate genes potentially associated with the distinctive shank phenotype of DT chickens. Therefore, both structural variant analysis and association mapping independently point toward biological mechanisms related to skeletal growth, connective tissue organization, and morphogenetic regulation. This convergence strengthens the hypothesis that the giant shank phenotype of DT chickens is not determined by a single isolated gene, but rather by a complex genomic architecture involving multiple developmental and structural pathways.

Future studies based on high-coverage whole-genome sequencing, transcriptome profiling of limb tissues, and functional validation experiments will be necessary to identify the causal mutations underlying the phenotype.

### 4.2. Limitations and Considerations

A limitation of the present study is the relatively modest sample size, which reflects the rarity and conservation status of the DT breed, although several studies on local or endangered livestock populations have been conducted using comparable or even smaller sample sizes [18,52,53]. Reduced sample sizes may decrease statistical power and increase the risk of both false-positive and false-negative associations in the GWAS. Nevertheless, several aspects strengthen the robustness and biological plausibility of our findings. First, association analyses were performed using a linear mixed model accounting for relatedness and population structure. Second, all candidate loci were supported by complementary analyses, including population differentiation (F_ST_), linkage disequilibrium patterns, QTL co-localization, and biological prioritization of candidate genes. GWASs performed on local or endangered chicken breeds frequently rely on relatively small sample sizes because of the limited availability of animals, yet they have successfully identified biologically meaningful loci. Moreover, a methodological limitation of the present study is the integration of high-density SNP-array data with genotypes derived from low-coverage whole-genome sequencing. Low sequencing depth can increase genotype uncertainty, particularly for heterozygous sites, and differences in genotype completeness between platforms may introduce residual bias. To minimize these effects, sequencing-derived variants were restricted to genomic positions corresponding to predefined array markers, unresolved allele inconsistencies were excluded, and identical post-merge quality-control criteria were applied to all samples and markers.

Finally, an additional limitation is the lack of an independent validation population. Although the candidate regions were consistently supported by complementary genomic analyses, including F_ST_, linkage disequilibrium characterization, QTL co-localization, and functional annotation, these approaches do not constitute independent replication. Therefore, the identified loci should be considered strong candidate regions associated with the giant shank phenotype rather than confirmed causal loci. Future studies in larger DT populations, preferably genotyped using a single platform and supported by detailed morphometric phenotyping, will be essential to validate these associations and refine the underlying causal variants.

Despite these limitations, the convergence of evidence from the mixed-model GWAS, LD structure, QTL co-localization, population differentiation, and biological annotation supports the identification of these regions as plausible candidate loci for the giant shank phenotype.

## 5. Conclusions

This study represents the first genome-wide investigation of the genetic basis underlying the giant shank phenotype in DT chickens. By integrating a GWAS, population differentiation analyses, LD patterns, QTL information, and candidate gene annotation, four genomic regions associated with this distinctive morphological trait were identified. Among these regions, the chromosome 2 locus containing *IGFBP1* and *IGFBP3* emerged as the strongest candidate, supported by the highest level of genetic differentiation between DT and control populations and by its co-localization with previously reported growth-related QTLs. Additional support was provided by loci containing *MYLK*, *CD44*, *KALRN*, and other genes involved in skeletal development, tissue remodeling, and cellular organization.

The integration of multiple independent genomic approaches consistently highlighted biological pathways related to growth regulation and skeletal development, suggesting that the giant shank phenotype may be influenced by a complex genetic architecture rather than by a single major-effect locus. These findings provide novel insights into the genomic basis of one of the most distinctive phenotypes observed in domestic chickens and establish a valuable framework for future studies. However, given the limited sample size, the integration of different genotyping platforms, and the lack of an independent validation population, the identified genomic regions should be regarded as candidate loci requiring confirmation in larger, independent populations before causal relationships can be established. Finally, further functional studies, including gene expression and experimental validation, will be required to determine the causal role of these candidate genes and pathways.

## Figures and Tables

**Table 1:** Summary of the four genomic regions significantly associated with the giant shank phenotype in Dong Tao chickens. For each candidate locus, the table reports chromosome position, association strength, genomic interval considered for annotation, number of SNPs within the region, genes located inside the candidate interval, and genes located closest to the association signal.

Region ID	Chr	Position (bp)	Lead −log_10_(P)	Window Start (bp)	Window End (bp)	SNPs (n)	Genes	Nearest Genes
Region_1	4	10,320,495	7.69	9,820,495	10,820,495	95	*GABRA3*; *GABRQ*; *SLITRK4*	*GABRQ*; *SLITRK4*; *GABRA3*; *GABRE*; *CNGA2*
Region_2	5	18,842,245	7.63	18,342,245	19,342,245	79	*APIP*; *CAPRIN1*; *CAT*; *CD44*; *EHF*; *ELF5*; *ENSGALG00000007886*; *ENSGALG00000011609*; *ENSGALG00000040371*; *ENSGALG00000040415*; *ENSGALG00000047061*; *ENSGALG00000049369*; *ENSGALG00000050964*; *ENSGALG00000052396*; *FBXO3*; *LMO2*; *NAT10*; *PDHX*; *SHANK2*; *SLC1A2*	*ENSGALG00000047061*; *ENSGALG00000011609*; *ENSGALG00000052396*; *NAT10*; *CAT*
Region_3	7	27,352,455	7.21	26,852,455	27,852,455	99	*ADCY6*; *CCDC14*; *ENSGALG00000050781*; *HACD2*; *KALRN*; *MYLK*; *PDIA5*; *SEC22A*	*MYLK*; *CCDC14*; *KALRN*; *ENSGALG00000050781*; *HACD2*
Region_4	2	55,145,792	6.73	54,645,792	55,645,792	117	*ADCY1*; *ENSGALG00000031260*; *ENSGALG00000035050*; *ENSGALG00000039589*; *ENSGALG00000045953*; *ENSGALG00000047009*; *ENSGALG00000049376*; *ENSGALG00000053689*; *IGFBP1*; *IGFBP3*; *NFX1*; *SLC12A7*; *TNS3*; *TPPP*; *TRIP13*	*TNS3*; *ENSGALG00000039589*; *ENSGALG00000049376*; *ENSGALG00000035050*; *ENSGALG00000047009*

**Table 2:** Positional candidate genes identified within the four GWAS-associated regions for the giant shank phenotype in Dong Tao chickens.

Region ID	SNP Position	Gene	Gene ID	Gene Start	Gene End	Distance to SNP (bp)	Priority Flag
Region_1	4:10,320,495	GABRQ	ENSGALG00000033183	10,644,261	10,711,020	323,766	Candidate by proximity
Region_1	4:10,320,495	SLITRK4	ENSGALG00000007242	9,901,515	9,904,127	416,368	Candidate by proximity
Region_1	4:10,320,495	GABRA3	ENSGALG00000007269	10,750,330	10,800,416	429,835	Candidate by proximity
Region_2	5:18,842,245	ENSGALG00000047061	ENSGALG00000047061	18,818,960	18,869,117	0	Candidate by proximity
Region_2	5:18,842,245	ENSGALG00000011609	ENSGALG00000011609	18,899,233	18,904,487	56,988	Candidate by proximity
Region_2	5:18,842,245	ENSGALG00000052396	ENSGALG00000052396	18,776,803	18,784,074	58,171	Candidate by proximity
Region_2	5:18,842,245	NAT10	ENSGALG00000014573	18,749,501	18,771,124	71,121	Candidate by proximity
Region_2	5:18,842,245	CAT	ENSGALG00000030187	18,920,696	18,939,330	78,451	Candidate by proximity
Region_2	5:18,842,245	CAPRIN1	ENSGALG00000013532	18,710,570	18,743,777	98,468	Candidate by proximity
Region_2	5:18,842,245	ELF5	ENSGALG00000035867	18,954,568	18,964,262	112,323	Candidate by proximity
Region_2	5:18,842,245	EHF	ENSGALG00000007817	18,995,653	19,029,466	153,408	Candidate by proximity
Region_2	5:18,842,245	LMO2	ENSGALG00000029892	18,658,103	18,665,579	176,666	Candidate by proximity
Region_2	5:18,842,245	FBXO3	ENSGALG00000034029	18,629,829	18,649,356	192,889	Candidate by proximity
Region_2	5:18,842,245	ENSGALG00000040371	ENSGALG00000040371	18,622,009	18,627,613	214,632	Candidate by proximity
Region_2	5:18,842,245	APIP	ENSGALG00000031286	19,085,949	19,098,880	243,704	Candidate by proximity
Region_2	5:18,842,245	PDHX	ENSGALG00000033753	19,099,003	19,134,736	256,758	Candidate by proximity
Region_2	5:18,842,245	ENSGALG00000049369	ENSGALG00000049369	18,572,412	18,582,854	259,391	Candidate by proximity
Region_2	5:18,842,245	ENSGALG00000040415	ENSGALG00000040415	18,554,978	18,570,650	271,595	Candidate by proximity
Region_2	5:18,842,245	CD44	ENSGALG00000039080	19,191,268	19,248,208	349,023	Candidate by proximity
Region_2	5:18,842,245	ENSGALG00000050964	ENSGALG00000050964	18,466,995	18,477,104	365,141	Candidate by proximity
Region_2	5:18,842,245	SLC1A2	ENSGALG00000037556	19,262,646	19,335,831	420,401	Candidate by proximity
Region_2	5:18,842,245	SHANK2	ENSGALG00000035916	18,179,810	18,393,764	448,481	Candidate by proximity
Region_2	5:18,842,245	ENSGALG00000007886	ENSGALG00000007886	19,338,964	19,346,387	496,719	Candidate by proximity
Region_3	7:27,352,455	MYLK	ENSGALG00000011708	27,172,896	27,356,465	0	Candidate by proximity
Region_3	7:27,352,455	CCDC14	ENSGALG00000044814	27,378,340	27,386,680	25,885	Candidate by proximity
Region_3	7:27,352,455	KALRN	ENSGALG00000011744	27,415,443	27,857,542	62,988	Candidate by proximity
Region_3	7:27,352,455	ENSGALG00000050781	ENSGALG00000050781	27,137,619	27,188,090	164,365	Candidate by proximity
Region_3	7:27,352,455	HACD2	ENSGALG00000011704	27,151,313	27,170,993	181,462	Candidate by proximity
Region_3	7:27,352,455	ADCY6	ENSGALG00000031244	26,948,199	27,144,911	207,544	Candidate by proximity
Region_3	7:27,352,455	SEC22A	ENSGALG00000011697	26,927,568	26,942,616	409,839	Candidate by proximity
Region_3	7:27,352,455	PDIA5	ENSGALG00000011689	26,824,064	26,916,163	436,292	Candidate by proximity
Region_4	2:55,145,792	TNS3	ENSGALG00000039139	55,050,336	55,235,133	0	Candidate by proximity
Region_4	2:55,145,792	ENSGALG00000039589	ENSGALG00000039589	55,305,607	55,312,884	159,815	Candidate by proximity
Region_4	2:55,145,792	ENSGALG00000049376	ENSGALG00000049376	54,976,627	54,977,754	168,038	Candidate by proximity
Region_4	2:55,145,792	ENSGALG00000035050	ENSGALG00000035050	55,319,904	55,333,431	174,112	Candidate by proximity
Region_4	2:55,145,792	ENSGALG00000047009	ENSGALG00000047009	55,333,143	55,347,701	187,351	Candidate by proximity
Region_4	2:55,145,792	IGFBP3	ENSGALG00000037940	54,930,529	54,947,966	197,826	High biological relevance
Region_4	2:55,145,792	ENSGALG00000045953	ENSGALG00000045953	55,351,276	55,354,731	205,484	Candidate by proximity
Region_4	2:55,145,792	NFX1	ENSGALG00000043724	55,368,341	55,428,626	222,549	Candidate by proximity
Region_4	2:55,145,792	IGFBP1	ENSGALG00000043493	54,916,522	54,922,765	223,027	High biological relevance
Region_4	2:55,145,792	ADCY1	ENSGALG00000031170	54,710,632	54,882,357	263,435	Candidate by proximity
Region_4	2:55,145,792	SLC12A7	ENSGALG00000042396	55,454,629	55,529,287	308,837	Candidate by proximity
Region_4	2:55,145,792	TRIP13	ENSGALG00000033834	55,539,174	55,554,509	393,382	Candidate by proximity
Region_4	2:55,145,792	ENSGALG00000031260	ENSGALG00000031260	55,554,625	55,556,797	408,833	Candidate by proximity
Region_4	2:55,145,792	ENSGALG00000053689	ENSGALG00000053689	55,559,010	55,576,761	413,218	Candidate by proximity
Region_4	2:55,145,792	TPPP	ENSGALG00000042784	55,608,568	55,657,394	462,776	Candidate by proximity

**Table 3:** Overlap between the candidate genomic regions identified in the present study and Chicken QTLdb.

Region ID	Chr	SNP Position (bp)	Window Start (bp)	Window End (bp)	QTL ID	Trait	QTL Start	QTL_End
Region_1	4	10,320,495	9,820,495	10,820,495	297,204	Shank length QTL	10,423,566	10,423,570
Region_1	4	10,320,495	9,820,495	10,820,495	214,415	Egg weight QTL	10,606,731	10,606,735
Region_2	5	18,842,245	18,342,245	19,342,245	139,665	Feed conversion ratio QTL	18,756,143	18,756,147
Region_2	5	18,842,245	18,342,245	19,342,245	139,577	Feed conversion ratio QTL	18,757,452	18,757,456
Region_4	2	55,145,792	54,645,792	55,645,792	153,737	Body weight QTL	55,584,939	55,584,943

## Data Availability

The datasets used in the current study have been deposited in the Research Data UNIPD Archive (https://researchdata.cab.unipd.it/id/eprint/1849 (accessed on 20 July 2026), DOI: 10.25430/researchdata.cab.unipd.it.00001849).

## Supplementary Materials

The following supporting information can be downloaded at: https://www.mdpi.com/article/10.3390/genes17080855/s1, Figure S1: Quantile–quantile plot of GWAS association statistics; Figure S2: Maximum F_ST_ values observed within the four candidate genomic regions; Table S1: Top 200 SNPs identified by the GWAS analysis; Table S2: Annotated genes located within the candidate genomic region; Table S3: Functional classification of candidate genes; Table S4: F_ST_ statistics calculated within the four GWAS candidate regions between DT chickens and the pooled control populations.

## Abbreviations

The following abbreviations are used in this manuscript:

AC	Ac chicken breed
*ADCY1*	Adenylate Cyclase 1
*ADCY6*	Adenylate Cyclase 6
*APIP*	APAF1-Interacting Protein
*CAT*	Catalase
*CAPRIN1*	Cell Cycle-Associated Protein 1
*CCDC14*	Coiled-Coil Domain-Containing 14
*CD44*	CD44 Molecule
CI	Choi chicken breed
DT	Dong Tao chicken breed
*EHF*	ETS Homologous Factor
*ELF5*	E74 Like ETS Transcription Factor 5
*FBXO3*	F-Box Protein 3
F_ST_	Fixation Index
*GABRA3*	Gamma-Aminobutyric Acid Type A Receptor Subunit Alpha3
*GABRQ*	Gamma-Aminobutyric Acid Type A Receptor Subunit Theta
GWAS	Genome-Wide Association Study
*HACD2*	3-Hydroxyacyl-CoA Dehydratase 2
HO	Ho chicken breed
HSP	Heat Shock Protein
*IGF*	Insulin-Like Growth Factor
*IGFBP1*	Insulin-Like Growth Factor Binding Protein 1
*IGFBP3*	Insulin-Like Growth Factor Binding Protein 3
*KALRN*	Kalirin RhoGEF Kinase
LD	Linkage Disequilibrium
*LMO2*	LIM Domain Only 2
LRT	Likelihood Ratio Test
MDS	Multidimensional Scaling
*MYLK*	Myosin Light-Chain Kinase
*NAT10*	N-Acetyltransferase 10
*NFX1*	Nuclear Transcription Factor X-Box Binding 1
*PDHX*	Pyruvate Dehydrogenase Complex Component X
*PDIA5*	Protein Disulfide Isomerase Family A Member 5
QTL	Quantitative Trait Locus
*SEC22A*	SEC22 Homolog A
*SHANK2*	SH3 and Multiple Ankyrin Repeat Domains 2
*SLITRK4*	SLIT and NTRK Like Family Member 4
*SLC1A2*	Solute Carrier Family 1 Member 2
*SLC12A7*	Solute Carrier Family 12 Member 7
SNP	Single Nucleotide Polymorphism
*TNS3*	Tensin 3
*TPPP*	Tubulin Polymerization-Promoting Protein
*TRIP13*	Thyroid Hormone Receptor Interactor 13
